# Advancing evidence-based regulation: Organization for the Study of Sex Differences and Society for Women’s Health Research support FDA action on menopausal hormone therapy and encourage broader sex-informed drug label updates

**DOI:** 10.1186/s13293-026-00838-4

**Published:** 2026-01-25

**Authors:** Rebecca L. Cunningham, Liisa A.M. Galea, Syreen Goulmamine, Georgios Kararigas, Kathryn G. Schubert, Kristen L. Zuloaga, Sofia B. Ahmed

**Affiliations:** 1 Department of Pharmaceutical Sciences College of Pharmacy, University of North Texas Health Science Center, TX Fort Worth, USA; 2 Organization for the Study of Sex Differences, Bandera, TX USA; 3 Centre for Addiction and Mental Health, Treliving Family Chair in Women’s Mental Health, Toronto, Canada; 4 Department of Psychiatry, Temerty Faculty of Medicine, University of Toronto, Toronto, Ontario, Canada; 5 Editorial Board, Biology of Sex Differences, New York City, NY USA; 6 Society for Women’s Health Research, Washington, D.C USA; 7 Department of Basic and Clinical Sciences, Medical School, University of Nicosia, UNIC Athens, Athens, Greece; 8 Department of Neuroscience & Experimental Therapeutics, Albany Medical College, Albany, NY USA; 9 Department of Medicine, Faculty of Medicine and Dentistry, University of Alberta, Edmonton, Alberta, Canada; 10 Women and Children’s Health Research Institute, University of Alberta, Edmonton, Alberta, Canada

## Abstract

Menopause is an important life-stage transition with substantial implications for health and quality of life. Menopausal hormone therapy (HT) remains among the most effective treatments for menopausal symptoms, yet clinical uptake has varied markedly over time as evidence regarding benefits and risks has evolved. The Organization for the Study of Sex Differences and the Society for Women’s Health Research outline our support for the US Food and Drug Administration’s (FDA) removal of the “black box” warning on menopausal HT labels. This important shift in federal policy supports evidence-based menopause care and reflects an evolving, evidence-responsive regulatory practice. We further call for consistent, evidence-based FDA review of whether approved product labeling adequately incorporates sex-related differences in pharmacokinetics, pharmacodynamics, and adverse event profiles, with updates to indications, dosing, and warnings where supported by robust data.

## History of menopausal hormone therapy and women’s health

Menopause marks a significant transition in a woman’s life influencing biological [1], cardiovascular [2], and immunological [3] aging and the brain [4]; access to safe and effective treatments is critical to supporting both short-term quality of life and long-term health. More than 30 symptoms are associated with menopause and perimenopause, including hot flashes, sleep disturbances, weight gain, osteoporosis, vaginal dryness, joint pain, mood changes, depression, and cognitive difficulties [5]. Menopausal hormone therapy (HT), often referred to in the past as hormone replacement therapy (HRT), is one of the most effective treatments for many of these symptoms and has been recommended for decades by major medical societies as a first-line therapy [6–8]. Despite these benefits, the use of HT has fluctuated dramatically over time as scientific understanding of its risks and benefits has evolved.

In the 1990s, widespread use of HT was driven by numerous observational studies demonstrating significant beneficial health effects. Large meta-analyses reported that HT use was associated with a 35% reduction in coronary heart disease [9], a 25% reduction in hip fracture [9], and a 29% reduction in dementia risk [10]. These findings generated widespread enthusiasm and helped establish HT as a cornerstone of menopausal care. To rigorously evaluate these associations, several randomized controlled trials (RCTs) were initiated, including the landmark Women’s Health Initiative (WHI) trials, which enrolled postmenopausal women between the ages of 50 and 79 with (*n* = 16,608) [11] and without (*n* = 10,739) [12] a uterus. As the most commonly used HT in the US at the time [13], participants received either oral conjugated equine estrogens (CEE) [with medroxyprogesterone acetate (MPA) in those with a uterus] or placebo. An ancillary trial, the Women’s Health Initiative Memory Study (WHIMS), aimed to determine whether HT could prevent cognitive decline or dementia [14].

In 2002 and again in 2004, the WHI findings sent shock waves through both the medical and scientific communities and the general public. The trials were both stopped early after results indicated increased risks of coronary heart disease (CHD), breast cancer, stroke, pulmonary embolism, and dementia among participants receiving HT. Only hip fracture [11, 12, 14, 15] and colorectal cancer risks were reduced [11, 14, 15]. The Women’s International Study of long Duration Oestrogen after Menopause (WISDOM), a multicenter, randomized, placebo-controlled, double-blind trial conducted in the UK, Australia, and New Zealand that also used CEE plus MPA reported increased cardiovascular and thrombotic risk [16], even with lower participant enrolment after being stopped early in response to the WHI findings.

These findings conflicted sharply with prior observational data and resulted in substantial public concern. In 2003, the U.S. Food and Drug Administration (FDA) issued a “black box” warning, the agency’s strictest caution, directly linking HT to serious risks including cancer and cardiovascular events. These warnings significantly altered prescribing patterns and public perception, leading to a substantial and rapid decline in HT use and leaving many women without effective symptom relief [17].

Over the following two decades, researchers worked to reconcile disparities between observational studies and the WHI trials. While it remains possible that early harm was offset by later benefit in observational studies, multiple explanations have emerged, including the “timing hypothesis” or “critical window” theory [18], the healthy cell bias [19] and the importance of HT formulation, duration of exposure, and dose [20]. The “critical window” framework proposes that HT is more beneficial when initiated close to the onset of menopause but may pose risks when started years later. Reanalysis of WHI data supported this hypothesis: women who initiated HT at younger ages (e.g., 50–59 years of age) experienced reductions in myocardial infarction, death, and overall adverse events [21]. A Danish open-label RCT enrolled 1,006 healthy recently menopausal women (mean age 50 years and, on average, seven months since menopause) and randomized participants to HT (triphasic estradiol plus norethisterone acetate, or estradiol alone in those without a uterus) or no treatment [22]. After 10 years, HT was associated with a significantly lower risk of the composite outcome of death, heart failure, or myocardial infarction with no increase in cancer, venous thromboembolism, or stroke. Similarly, an Estonian RCT in healthy menopausal participants showed no effect of CEE and MPA on coronary and cerebrovascular outcomes or breast cancer, but a decrease in fractures in HT users compared to non-users after adjustment for age at recruitment and former oral contraceptive use [23].

Observational studies also reinforced the timing effect, at least as it related to cardiovascular and cognitive health. For example, a reanalysis of the Nurses’ Health Study demonstrated that women beginning HT near menopause had a significantly reduced risk of CHD, with no significant relation between HT and CHD among women who initiated therapy at least 10 years after menopause [24]. Furthermore, the Cache County Study found that HT initiated within five years of menopause reduced the incidence of Alzheimer's disease by 30% whereas initiation more than five years after menopause offered no reduction in risk [25].

Pre-clinical research further supported timing of HT initiation as a key determinant of the effects of therapy. Studies in rodent models mimicking menopause demonstrated that immediate post-menopause estradiol therapy reduced inflammation and protected the brain after stroke, whereas delayed therapy showed no such benefits [26–28]. Further, the duration of time spent within an estrogen-deficient state prior to HT exposure has been associated with worse stroke outcomes [29, 30]. These studies highlight that early HT intervention may be better than later HT interventions, supporting the “timing hypothesis” or “critical window” theory as it relates to cardiovascular and cognitive health. However, an individual participant meta-analysis reported that the relative risks for breast cancer in current systemic HT users were greater if therapy was begun before or soon after menopause than after a longer gap [31], underscoring the importance of accounting for differing person-level factors when considering HT use.

There is also evidence that HT may have differential effects based on the health of the individual. In the WHI, exclusion criteria were limited, such that women living with hypertension, diabetes and higher BMI were eligible and included in the trial, and it is possible that the impacts of HT differed according to underlying health status, although this was not specifically studied. Mechanistic research introduced the concept of “healthy cell bias,” suggesting that estradiol loses its protective effects when target tissues have advanced too far along a pathological trajectory [19, 32]. Thus, estrogens can be protective in healthy cells, but the same formulations may be detrimental when disease pathology is already present [33, 34].

The HT formulation in the WHI is also an important factor that can help explain variation in risk profiles. The WHI used CEE, in which estrone is the predominant estrogen with ~ 0.5% estradiol [35]. Both these circulating estrogens decline at menopause but estradiol more so than estrone, and estradiol is the more potent of the estrogens [36]. Most contemporary HT incorporates 17β estradiol, a form supported by decades of research demonstrating cardiovascular, skeletal, and neuroprotective benefits [37]; a meta-analysis showed that HTs containing CEE were associated with less cognitive benefit than estradiol-based therapies [38, 39]. Route of administration matters as well: oral HT undergoes first-pass liver metabolism and is linked to a higher risk of venous thromboembolism [40–43], loss of kidney function [44], increased blood pressure [45] and hypertension [46] than transdermal formulations. In a large observational study, transdermal vaginal formulations were associated with risk reductions in cardiovascular disease, cancer, and mortality [47]; another study reported positive associations with transdermal estradiol compared to oral estradiol on episodic memory, the form of memory initially affected in Alzheimer’s disease [48]. However, some observational studies may be affected by “healthy user bias,” as women who choose HT often enjoy better access to healthcare and overall health status, which may be particularly prominent in countries without free access to healthcare [49]. However, the reverse could also be true, as women who are prescribed HT in observational studies may be more affected by menopausal symptoms and have poorer health-related quality of life [50].

To address these questions directly, multiple RCTs have been conducted [51–55] including the Kronos Early Estrogen Prevention Study (KEEPS) [51] and the Early versus Late Intervention Trial with Estradiol (ELITE) [53] trials. The KEEPS study was a multicenter, randomized, double-blind, placebo-controlled trial comparing oral CEE with transdermal estradiol in healthy women within three years of menopause and was designed to test whether starting HT soon after menopause slowed early atherosclerosis progression. Over 4 years, neither form of estrogen changed carotid artery intima-media thickness (CIMT) or coronary artery calcification progression compared with placebo, although beneficial effects on vasomotor symptoms were reported with both forms of estrogen use [51].

Ancillary KEEPS studies evaluated cognition, mood and brain MRI markers alongside vascular endpoints [55, 56] In KEEPS-Cog, HT initiation did not improve or worsen cognitive performance compared with placebo over 48 months. Low dose oral CEE was associated with modest improvements in mood, while transdermal estradiol was not [55]. Neuroimaging analyses also reported baseline imbalances across arms (e.g., higher white matter hyperintensity (WMH) burden and proportion of participants with a high genetic risk for late onset sporadic Alzheimer’s disease (APOE4 genotype) in the transdermal estradiol arm) and found that WMH increased over time. WMH accumulation was greater with oral CEE compared to placebo, while transdermal estradiol did not differ from placebo, and the two active arms did not differ significantly from each other [56]. No long-term effect of 4-years of menopausal HT was observed on white matter integrity when compared to placebo in the KEEPS Continuation study, consistent with accumulating evidence supporting the safety of short-term HT use in recently postmenopausal women [57].

The ELITE study [53] was an RCT designed to examine the vascular effects of early versus late menopausal HT with oral estradiol in healthy women. Oral estradiol therapy initiated within 6 years of menopause slowed CIMT progression, whereas initiation ≥ 10 years after menopause did not; estradiol had no effect on coronary CT measures of atherosclerosis compared to placebo. The ELITE cognitive ancillary trial reported that estradiol had no effect on cognitive performance compared to placebo [54], regardless of timing relative to menopausal onset.

While these RCTs use surrogate endpoints rather than hard clinical outcomes, they reinforce the importance of timing, formulation, and route in determining the safety and efficacy of HT. These sources of variation likely influence health outcomes in a genotype- and hormone sensitivity-dependent manner, highlighting the importance of personalized approaches in appropriately assessing the risks and benefits of HT.

## Support for FDA removal of the HT warning

Two decades of research from observational studies, clinical trials, and experimental investigations have clarified that the original WHI findings do not apply uniformly to all women or all HT; HT carries a much more nuanced risk profile than the 2003 “black box” warning suggested. Stratified data now demonstrate that age, time of HT initiation relative to menopause age, baseline health conditions, HT formulation, dosage, duration of use, and route of administration influence risk profiles in critical ways [58]. A growing body of evidence supports the safety of HT when used appropriately and in alignment with clinical guidance. For example, large cohort studies and a meta-analysis showed no evidence of risk of recurrence in breast cancer survivors with use of low-dose vaginal estrogen [59, 60]. Similarly, two large nested case-control studies reported that transdermal HT preparations were not associated with risk of venous thromboembolism, which was consistent across different regimens [61]. 

On November 10, 2025, the U.S. Department of Health and Human Services (HHS) and the FDA announced that the FDA would remove its broad “black box” warnings from HT products for menopause [62]. The FDA and HHS characterized the long-standing boxed warnings for menopausal HT as contributing to decades of “fear and misinformation” and to a “distortion of risk”. The agency has advised drug sponsors to remove references to elevated cardiovascular disease, breast cancer, and dementia risk from boxed warnings, while maintaining the endometrial cancer warning specific to systemic estrogen-alone therapies. Updated product labeling will also emphasize evidence-based prescribing guidance, including recommendations to initiate HT within 10 years of menopause onset or before age 60.

These changes reflect the strong scientific consensus that timing, formulation, route, and health status are key determinants of benefit and risk and demonstrate the FDA’s commitment to ensuring that menopausal HT information aligns with current science. Removal of the warning allows clinicians to better tailor therapy to each patient’s needs and ensures that individuals can make informed decisions based on accurate, updated information. Alongside labeling changes intended to better align benefit-risk communication with contemporary evidence, the FDA has also expanded the available approved hormonal and non-hormonal treatments for vasomotor symptoms [63].

The Organization for the Study of Sex Differences (OSSD) and the Society for Women’s Health Research (SWHR) strongly support the FDA’s action. Ensuring that drug labeling reflects modern scientific evidence is essential to improving access to effective menopausal care.

## Broader significance for women’s health

The FDA’s decision to remove outdated boxed warnings and update HT labeling represents an important shift in federal policy supporting evidence-based menopause care. All female individuals will eventually experience menopause, whether it occurs naturally or is induced surgically or through medical treatment, and in some cases, it may occur early or prematurely.

Menopausal symptoms can persist for a decade or longer [64] and contribute to significant impacts on workplace participation, chronic disease risk, intimate relationships, and overall quality of life [65]. Yet for more than 20 years, policy driven by misinterpreted data has discouraged appropriate use of HT, contributing to both misconceptions and undertreatment [66]. Updated regulatory guidance ensures that national medication policy is better aligned with current science, reducing outdated barriers that have limited women’s access to effective care.

Accurate labeling also strengthens shared decision-making by ensuring clinicians can discuss HT confidently and patients can evaluate treatment options without outdated safety fears [67]. This is especially important given the long-standing stigma surrounding menopause and the limited training many healthcare providers receive in menopause management [68]. Nearly 85% of midlife women experience menopause-related symptoms, many of which have significant occupational consequences [69–71]. Initial findings from the Society for Women’s Health Research EMPACT Menopause Study emphasize this impact, showing that women’s most disruptive symptoms include sleep disturbances, cognitive challenges, such as brain fog and difficulty concentrating, changes in weight or body shape, and mood fluctuations that they feel impact their workplace performance [72]. Studies support the role of HT in effectively and safely treating these symptoms. By addressing these obstacles, women can continue to pursue their careers, engage at a high level, and contribute meaningfully to their workplace and society. Supporting women during the menopausal transition is vital for retaining talent, fostering career growth, and boosting economic development [73, 74].

Regulatory perspectives are increasingly recognizing that in this era of precision health, drug labeling must better reflect clinical heterogeneity and context-dependent risk-benefit profiles. For example, after publication of an RCT of men with hypogonadism and preexisting or at high risk of cardiovascular disease showing that testosterone gel was noninferior to placebo with respect to the incidence of major adverse cardiac events [75] and reviewing the results from required postmarket ambulatory blood pressure studies, the FDA recommended removing boxed-warning language about increased risk of major adverse cardiovascular outcomes and added stronger labeling about increased blood pressure across testosterone products [76]. On December 10, 2025, the FDA held an Expert Panel to discuss potential updates to testosterone labeling, including revisions to approved indications [77]. 

While not endorsing any specific policy approach, we support the principle of timely, evidence-responsive reassessment of approved indications and safety warnings as the scientific literature evolves, underscoring the centrality of rigorous science in regulatory decision-making. By modernizing HT regulation, the FDA underscores the national importance of addressing menopause as a public health priority. Continued monitoring, research investment, and policy alignment will be essential to ensure that the growing body of evidence consistently translates into real-world improvements in access and long-term health for millions of women.

Recent policy initiatives and research roadmaps emphasize reframing women’s health through a life-course lens that includes midlife and older age [78]. Menopause has been noted as underfunded relative to its clinical importance, and the National Institutes of Health has recently created a dedicated research category for menopause [79]. Further advancing women’s health priorities requires an evidence-based approach. This could include large pragmatic RCTs of both hormonal and non-hormonal therapies embedded in routine health systems with registry-based randomization, alongside rigorous “target-trial emulation” analyses using high-quality electronic health record and claims data to allow for long-term monitoring of important health outcomes. This work should be guided by strong preclinical and mechanistic studies clarifying how factors such as timing of initiation, route, dose, duration of use and progestogen type influence biological pathways so that safety communication shifts toward product-specific guidance rather than relying on broad class-wide statements.

## Action item: request for FDA review of Sex-Informed labeling

There are pronounced differences by sex in the response to pharmacotherapies [80–82] spanning drug efficacy, therapeutic thresholds, and adverse drug reactions (ADR). For example, based on demonstrated pharmacokinetic and safety data, the FDA reduced the recommended dose of zolpidem for women to 50% of that for men [83]. Similarly, a recent systematic review examining FDA-approved anticancer drugs identified major sex differences in pharmacokinetics, suggesting that sex-specific dosing strategies may enhance personalized treatment outcomes [84]. Worldwide, most ADR disproportionately affect female individuals of reproductive age, and more serious reactions are seen in male individuals [85], suggesting the importance of understanding drug reactions across sexes and ages. Recommendations based on sex-disaggregated data are vital to improving outcomes; by way of illustration, recent observational studies of guideline-recommended cardiovascular medications suggest differential harms and benefits based on sex, with lower survival in women compared to men for a given dose [86, 87].

Consistent, evidence-based regulatory review across therapeutic classes is of clear importance. Indications/contraindications, warning labels, and dosing guidance should be regularly reevaluated to ensure alignment with current science. Clear, current, and scientifically grounded labeling is essential to patient safety, informed decision-making, and maintaining public trust. An evidence-based approach explicitly incorporating sex and other sex-specific variables will ensure therapies are evaluated, labeled, and used safely and effectively, with better health outcomes for everyone.

## Data Availability

No datasets were generated or analysed during the current study.

## Abbreviations

ADR: Adverse Drug Reaction
BMI: Body Mass Index
CEE: Conjugated Equine Estrogen
CHD: Coronary Heart Disease
CIMT: Carotid Artery Intima-Media Thickness
ELITE: Early versus Late Intervention Trial with Estradiol
FDA: Food and Drug Administration
HHS: Health and Human Services
HRT: Hormone Replacement Therapy
HT: Hormone Therapy
KEEPS: Kronos Early Estrogen Prevention Study
MPA: Medroxyprogesterone Acetate
OSSD: Organization for the Study of Sex Differences
RCT: Randomized Controlled Trial
SWHR: Society for Women’s Health Research
WHI: Women’s Health Initiative
WHIMS: Women’s Health Initiative Memory Study
WMH: White Matter Hyperintensity
